# Identification of prognostic genes through expression differentiation during metastatic process in lung adenocarcinoma

**DOI:** 10.1038/s41598-017-11520-6

**Published:** 2017-09-11

**Authors:** Ning An, Xue Yang

**Affiliations:** Department of Oncology, The Affiliated Hospital of Qingdao University, Qingdao, 266003 China

## Abstract

Cancer is a highly complicated biological process due to large scale heterogeneity. Identification of differentially expressed genes between normal and cancer samples is widely utilized in the discovery of prognostic factors. In this study, based on RNA sequencing data of lung adenocarcinoma, we focused on the expression differentiation during *confined* (with neither lymph node invasion nor distant metastasis) primary tumors and *lymphnode* (with only lymph node invasion but not distant metastasis) primary tumors. The result indicated that differentially expressed genes during confined-lymphnode transition were more closely related to patient’s overall survival comparing with those identified from normal-cancer transition. With the aid of public curated biological network, we successfully retrieved the biggest connected module composed of 135 genes, of which the expression was significantly associated with patient’s overall survival, confirmed by 9 independent microarray datasets.

## Introduction

Due to the rapid development of molecular cancer research, more and more prognostic factors and therapeutic targets have been discovered, greatly aiding the clinical cancer treatment^1, 2^. However, carcinogenesis is still a highly complicated process, containing substantial and extensive molecular dysregulations. Although remarkable progress has been made to investigate the underlying molecular mechanism, the understanding of the complicated carcinogenesis process was enormously hindered by large-scale tumor heterogeneity^3–8^, adding to the difficulty of molecular cancer research. Therefore, understanding the intricate underlying molecular mechanisms is essentially important in introducing prognostic markers and potential therapeutic targets.

The comparison between cancer samples and normal tissues has been repetitively and effectively utilized in many studies^9–12^. Certainly, as for the process of carcinogenesis, normal tissue is the start point, whereas cancer tissue is the end event of the whole process. Therefore, the differentially expressed genes (DEGs) between normal and cancer samples might contain substantial information of cancer transformation and clinical prognosis. However, the normal-cancer comparison might be oversimplified by only looking into the two extremes of carcinogenesis, and focusing upon specific biological event after cancer transformation is complete might also be informative of patient’s prognosis.

Lymph node and distant metastasis is the main cause of treatment failure and the mortality of cancer patients. In most cancers, including lung adenocarcinoma (LUAD), the first site of tumor metastasis is lymph nodes, and lymph node invasion is unquestionably one of major criteria for evaluating patient prognosis and further options of clinical implementations^13, 14^. Moreover, lymph node invasion, mostly as the first step of distant metastasis in LUAD, has proven to be an independent predictor of poor outcome in numerous solid organ tumors, including invasive breast carcinoma^15^, prostatic adenocarcinoma^16^, sporadic colorectal cancer^17^, as well as lung cancer^18^. Admittedly, each step of metastatic process might be regarded as a potential target for the anti-tumor therapy, and understanding the metastatic process is probably a very promising maneuver for discovering potential therapeutic targeting^19^. Therefore, DEGs identified between different metastatic stages might also be helpful in discovering prognostic molecules.

The Cancer Genome Atlas (TCGA) database was a powerful resource of genomic data of various cancers, greatly helpful in the discovery of prognostic signature. For instance, Shukla *et al*. successfully retrieved a four-gene signature statistically significantly stratified overall survival of LUAD patinets in important clinical subsets, including Stage I and EGFR wild-type, and EGFR mutant subgroups^20^. In this study, we used LUAD RNA sequencing (RNA seq) data downloaded from TCGA database. Instead of only focusing on normal-cancer comparison, we divided the primary tumor samples into three groups according to metastatic status: (1) *confined*: primary tumors with neither lymph node invasion nor distant metastasis; (2) *lymphnode*: primary tumors with only lymph node invasion but not distant metastasis; (3) *metastasis*: primary tumors with distant metastasis. Therefore, since lymph node invasion and distant metastasis were greatly important in prognosis prediction and clinical implementations, the identification of DEGs during confined-lymphnode and lymphnode-metastasis transition might be a novel channel to understand the underlying mechanism of tumor invasion and migration. Hopefully, this study might provide another perspective in discovering promising prognostic factors and therapeutic targets for LUAD patients.

## Method

### Data retrieval

The transcriptional expression profile of LUAD was downloaded from TCGA data portal “RTCGA.rnaseq” (http://rtcga.github.io/RTCGA), containing 515 cancer samples and 59 normal tissues. One hundred and sixteen paired samples (including 58 cancer samples and 58 corresponding adjacent normal tissues) were retrieved to reduce potential individual bias in differentially expressed gene (DEG) identification. Moreover, three types of primary tumors were also collected to identify DEGs between each two sequential time points, including 218 confined samples, 124 lymphnode sample and 22 metastasis samples. Additionally, overall survival (OS) and other clinicopathological informations of cancer patients were also obtained from TCGA data portal “RTCGA.rnaseq”. As for microarray data validation, 9 LUAD microarray datasets were downloaded from Gene Expression Omnibus (GEO) database, with GEO accession numbers GEO68571 (n = 86), GEO68465 (n = 443), GEO41271 (n = 181), GEO11969 (n = 90), GEO30219 (n = 85), GEO42172 (n = 133), GEO50081 (n = 127), GEO13213 (n = 117), and GEO8894 (n = 62).

### Data prepossessing and normalization

The *log2* transformed normalized RNAseq counts [using RNASeq by Expectation Maximization (RSEM) method] of LUAD were retrieved for further analysis. Genes with missing values in more than half of all the subgroup samples were all eliminated from further analysis, and all the missing values were imputed through Bioconductor package “impute”. Additionally, the whole TCGA transcriptional profile was normalized using “Cyclicloess” method with “limma” R package to further approximate normal distribution. As for GEO microarray datasets, all the normalized expression profiles and clinical information were downloaded directly from GEO database.

### Calculation of differential score in each transition

Due to the limited sample numbers, we considered both fold change and the *p* value of Wilcoxon rank sum and signed rank test (Wilcox test) to calculate differential score. Differential score *D*_*i*_ for gene *i* was calculated as follows:

1 $${D}_{i}={{\rm{l}}{\rm{o}}{\rm{g}}}_{2}(F{C}_{i})\,\ast \,(-{{\rm{l}}{\rm{o}}{\rm{g}}}_{10}({P}_{i}))$$

In which, *FC*_*i*_ represented the fold change of gene *i*, and *P*_*i*_ represented the *p* value of Wilcox test.

### Establish merged biological network

The protein–protein interaction network was downloaded from the Human Protein Reference Database (HPRD), and Kyoto Encyclopedia of Genes and Genomes (KEGG) network. Therefore, gene regulatory network was established by merging the two networks, including 10,340 nodes and 60,642 edges after eliminating self-loops and duplicated edges.

### Survival analysis

Principal component analysis (PCA) is a statistical procedure using an orthogonal transformation to convert a set of observations into a set of linearly uncorrelated variables, of which the first principal component (PC1) has the largest possible variance. Therefore, calculated PC1 value of the candidate genes across the expression profile of cancer patients, and then all the cancer patients were divided into two groups according to the median of PC1 value. In this way, the expression of these candidate genes were linearly transformed into PC1 values capturing the largest variance of these genes’ molecular characteristics. Kaplan–Meier survival analysis and the log-rank test were used to evaluate the prognostic difference between the two PC1-assigned groups. The Cox proportional hazards regression model was used to evaluate the independence of the prognostic factors in a stepwise manner. Samples in the each dataset with complete information of age, sex, stage, and OS were used for Cox analysis, and a value of *p* < 0.05 was regarded as significant.

## Results

### Expression differentiation between consecutive metastatic time points were not as distinct as normal-cancer paired samples

**Figure 1:**
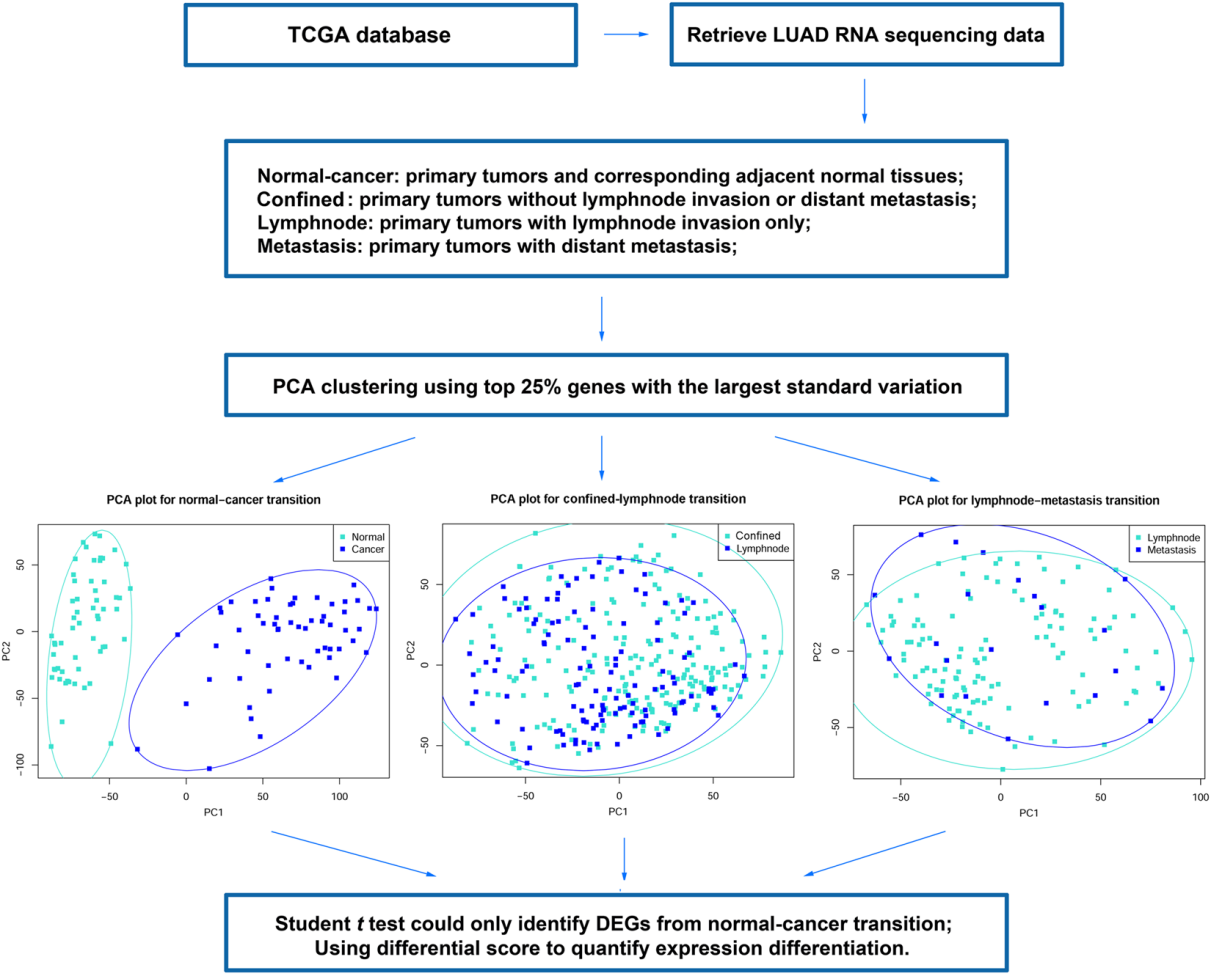
Schematic diagram of the reason why use differential score to identify DEGs during metastasis.

In order to identify significant DEGs, paired *t* test was used to identify DEGs in normal-cancer paired samples, and unpaired Student’s *t* test was conducted between each pair of consecutive metastatic time points. Furthermore, *p* values of *t* test were also adjusted using false discovery rate (*FDR*) method. The result indicated that 5405 genes were significantly differentiated in normal-cancer transition, including 2676 up-regulated and 2729 down-regulated genes (*FDR* < 0.001). However, no significant DEGs were identified in neither confined-lymphnode nor lymphnode-metastasis transition (*FDR* < 0.001). In addition, principle component analysis (PCA) of the normal-cancer, confined-lymphnode and lymphnode-metastasis data were conducted with corresponding top 25% genes with the largest standard deviation in each dataset. In normal-cancer paired data, the cancer samples and normal samples could be clustered into two distinct groups, while in the other two transitions, the cancer samples and normal samples were just dispersing randomly within each other (Fig. 1), suggesting the traditional *t* test was not suitable for DEG identification in these data without drastic expression differentiation.

### Expression differentiation in confined-lymphnode transition was more relevant to survival status

**Figure 2:**
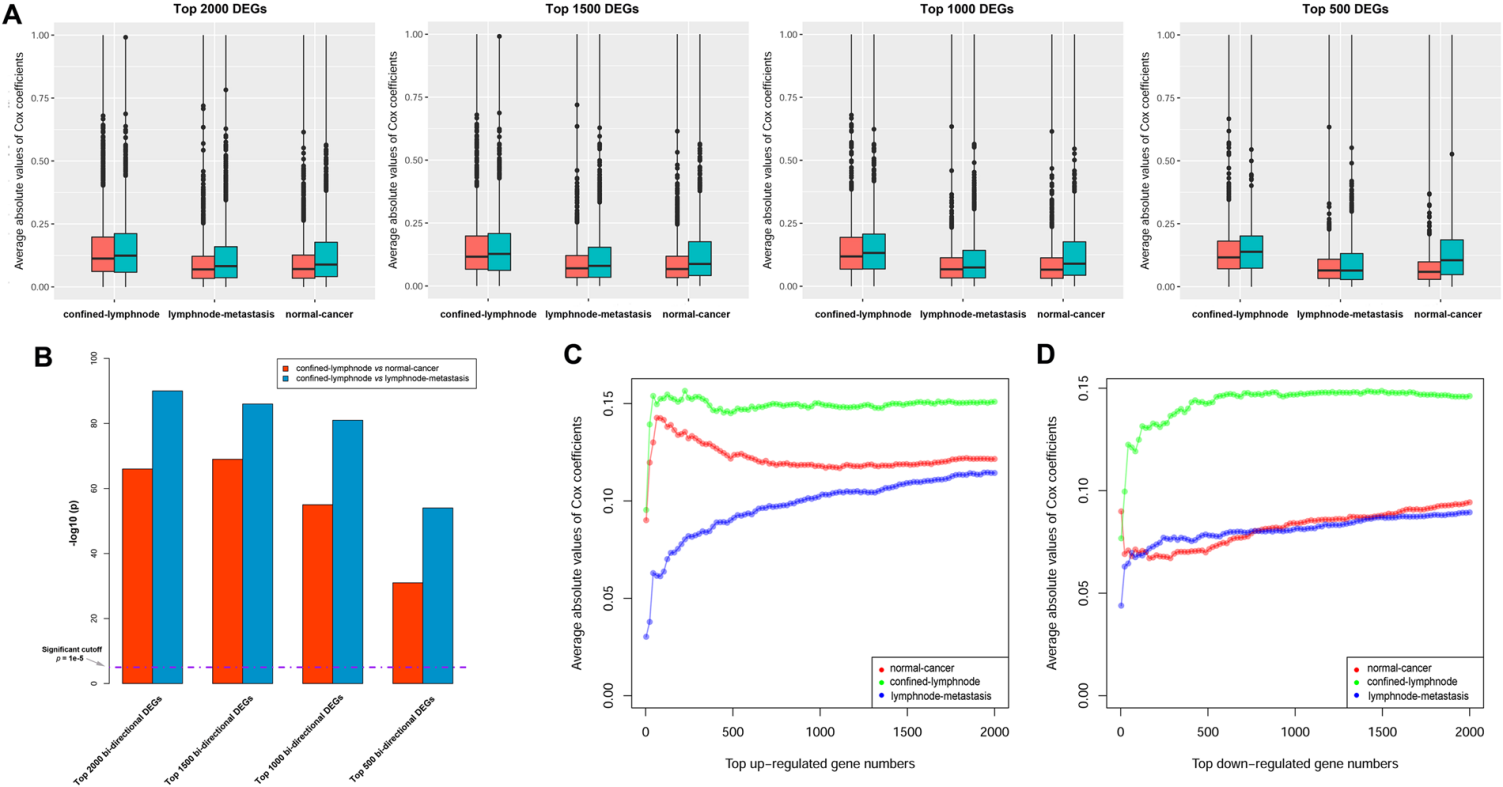
DEGs identified from confined-lymphnode transition contained the most prognostic information. (**A**) Top 2000, 1500, 1000 and 500 up-regulated and down-regulated were collected to calculate the average absolute values of Cox correlation coefficients. Red bar represented up-regulated DEGs, while green bar represented down-regulated ones. (**B**) Corresponding *p* values of average absolute values in each transition. The average values of top bi-directional DEGs (e.g. top 2000 bi-directional DEGs included 2000 up-regulated and 2000 down-regulated DEGs) were compared between DEGs identified during each transition using Wilcox test, and –log10 transformed *p* value were plotted against *y* axis. *P* value < 1e-05 was considered as significant. **(C**,**D)** Association between top DEG numbers and average Cox coefficients for both up-regulated DEGs (**C**) and down-regulated DEGs (**D**), respectively. *X* axis represented the top DEG numbers, and *y* axis represented the average absolute value of Cox coefficients of these DEGs.

Differential score (described in method section) denoted the status of expression differentiation, and the absolute value of Cox correlation coefficient quantified the interrelation between gene expression and patient’s OS. In order to compare the survival association of the DEGs in three transitions (normal-cancer, confined-lymphnode, and lymphnode-metastasis transition), we collected top 2000, 1500, 1000, and 500 up-regulated and down-regulated DEGs based on differential score in each transition, and calculated the average absolute values of Cox correlation coefficients to quantify the OS correlation of corresponding collected gene groups (Fig. 2A). The result indicated that the correlation between OS and DEGs in confined-lymphnode transition were all significantly higher than the other two transitions in both differential directions (Wilcox test, *p* < 1e-5), and corresponding *p* values were illustrated in Fig. 2B. Moreover, the association between average absolute Cox coefficient values (AACCV) and top gene numbers (TGN) was also illustrated in both up-regulated genes (Fig. 2C) and down-regulated genes (Fig. 2D), respectively. The curves in confined-lymphnode transition were located above those of the other two transitions in both differential directions, indicating that DEGs in confined-lymphnode transition were probably more relevant to patient’s OS with the comparison to those in the other two transitions. In order to complete the comparisons between each comparison among four sample types, we also conducted AACCV-TGN analysis in six comparisons (i.e. normal-confined, confined-lymphnode, lymphnode-metastasis, confined-metastasis, normal-lymphnode, and normal-metastasis) in both up-regulated and down-regulated directions (Supplementary Figure S1). The result indicated that the DEGs in confined-lymphnode transition performed best in both directions, indicating molecular dysregulation during confined-lymphnode transition probably contained much more prognostic information comparing with the others.

### Density plots of Cox correlation coefficients in each transition

**Figure 3:**
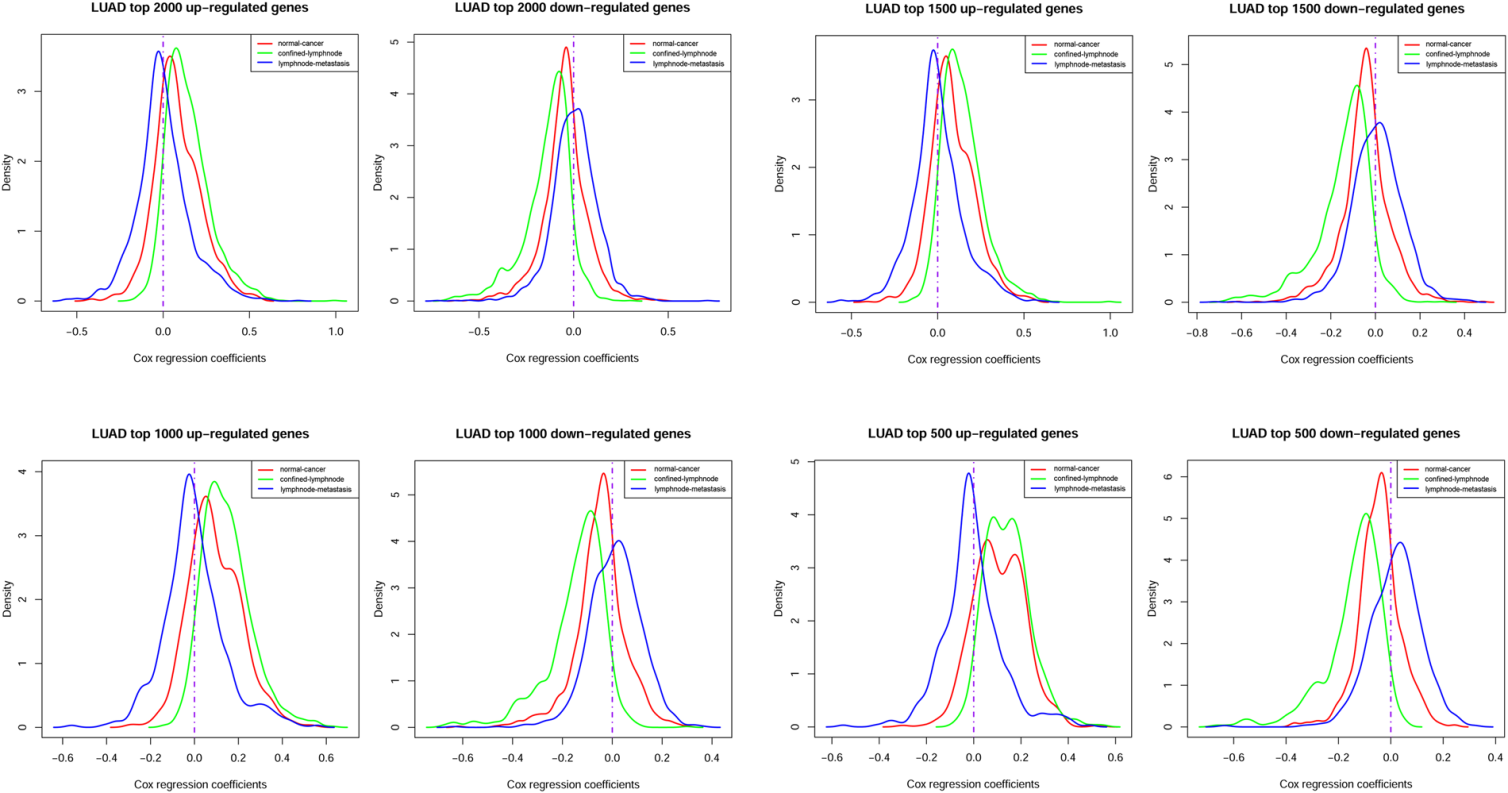
Density plot of Cox correlation coefficients within each DEG groups.

Top 2000, 1500, 1000, and 500 down-regulated and up-regulated genes were collected according to differential score in each transition, respectively. The density plots of Cox correlation coefficients were illustrated in Fig. 3, with red curves represented normal-cancer transition, green curves represented confined-lymphnode transition, and blue curves represent lymphnode-metastasis transition. The result revealed that the Cox coefficient density of top 2000 DEGs during three transitions were similarly distributed, i.e. normal distribution with the mean near 0. However, with fewer top genes we used, the density curve of Cox coefficients in confined-lymphnode transition were gradually separated from the others, suggesting a stronger OS association of these DEGs. Meanwhile, the mean Cox coefficients of up-regulated DEGs in confined-lymphnode transition was inclined to be > 0, and the mean of down-regulated DEGs was inclined to be < 0, indicating the overexpression of most of these up-regulated genes might probably shorten patient’s OS, while underexpression might suggest a better prognosis.

### Identify significant DEGs in three transitions

**Figure 4:**
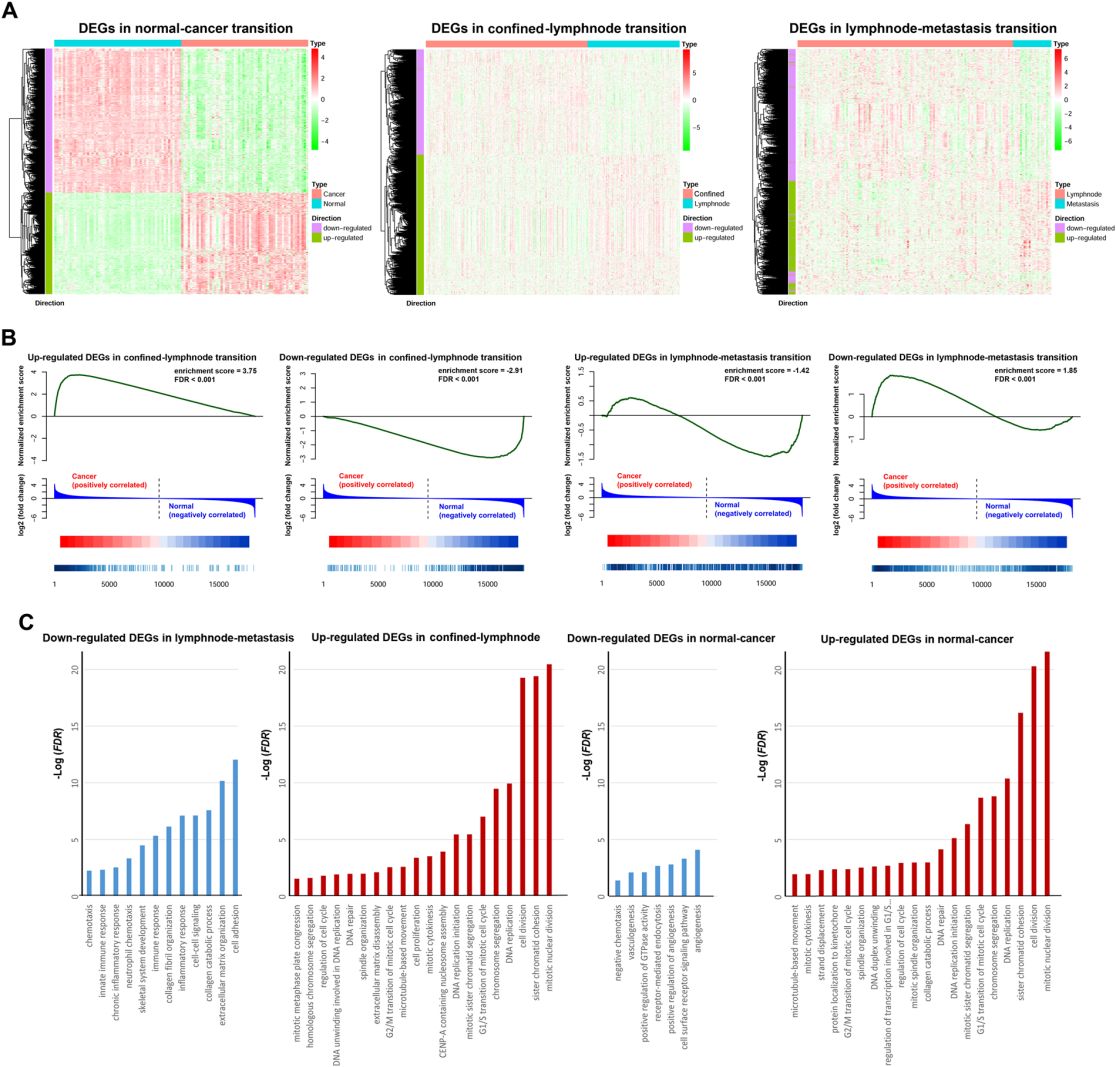
Identification of significant DEGs during each transition based on differential score. (**A**) Heat maps of corresponding significant DEGs identified in each transition. Columns represented samples, and rows represented genes. DEGs were also clustered using unsupervised clustering algorithm. (**B**) GSEA analysis of DEGs in confined-lymphnode and lymphnode-metastasis transition in normal-cancer paired data. (**C**) GO analysis of corresponding significant DEGs.

Kolmogorov-Smirnov test was conducted to prove that the differential scores in three transitions could significantly approximate normal distribution (normal-cancer: *D* = 0.221, *p* < 0.001; confined-lymphnode: *D* = 0.283, *p* < 0.001; lymphnode-metastasis: *D* = 0.317, *p* < 0.001). Therefore, one-sided normal distribution function was used to identify significant DEGs in corresponding normal distribution of each transition (*p* < 0.05). In this manner, 1216 DEGs were identified in normal-cancer paired data, including 502 up-regulated and 714 down-regulated DEGs; 1077 DEGs were found in confined-lymphnode transition, including 616 up-regulated and 461 down-regulated DEGs; and 1306 DEGs were identified during lymphnode-metastasis transition, including 530 up-regulated and 776 down-regulated DEGs (Fig. 4A). Since normal-cancer comparison has always been repetitively utilized in cancer research, gene set enrichment analysis (GSEA) was used to illuminate the distribution of DEGs identified from the other two transitions against normal-cancer paired data (Fig. 4B). The result indicated that the expression differentiation of confined-lymphnode DEGs were significantly accordant with those in normal-cancer transition. Although lymphnode-metastasis DEGs were also statistically significant, the normalized enrichment scores (NES) were quite lower than those of confined-lymphnode DEGs, and the differential direction was opposite to that in normal-cancer transition (Fig. 4B).

Gene ontology (GO) biological process enrichment analyses were carried out via the DAVID bioinformatics tool (http://david.abcc.ncifcrf.gov, Fig. 4C). GO analysis indicated that normal-cancer up-regulated DEGs were greatly related to cell division, cell cycle, and DNA replication, which was highly consistent with confined-lymphnode up-regulated DEGs. A very small number of GO terms were weakly enriched with normal-cancer down-regulated DEGs, including angiogenesis, cell surface receptor signaling pathway, and vasculogenesis, and the *FDR* values were relatively close to the verge of insignificance (with the criterion *FDR* = 0.05). No GO items were significantly enriched with confined-lymphnode down-regulated DEGs, while lymphnode-metastasis down-regulated DEGs were significantly enriched in cell adhesion, extracellular matrix organization and collagen catabolic process, highly related to invasion and migration process (Fig. 4C).

### DEGs during confined-lymphnode transition were most significantly correlated with OS

**Figure 5:**
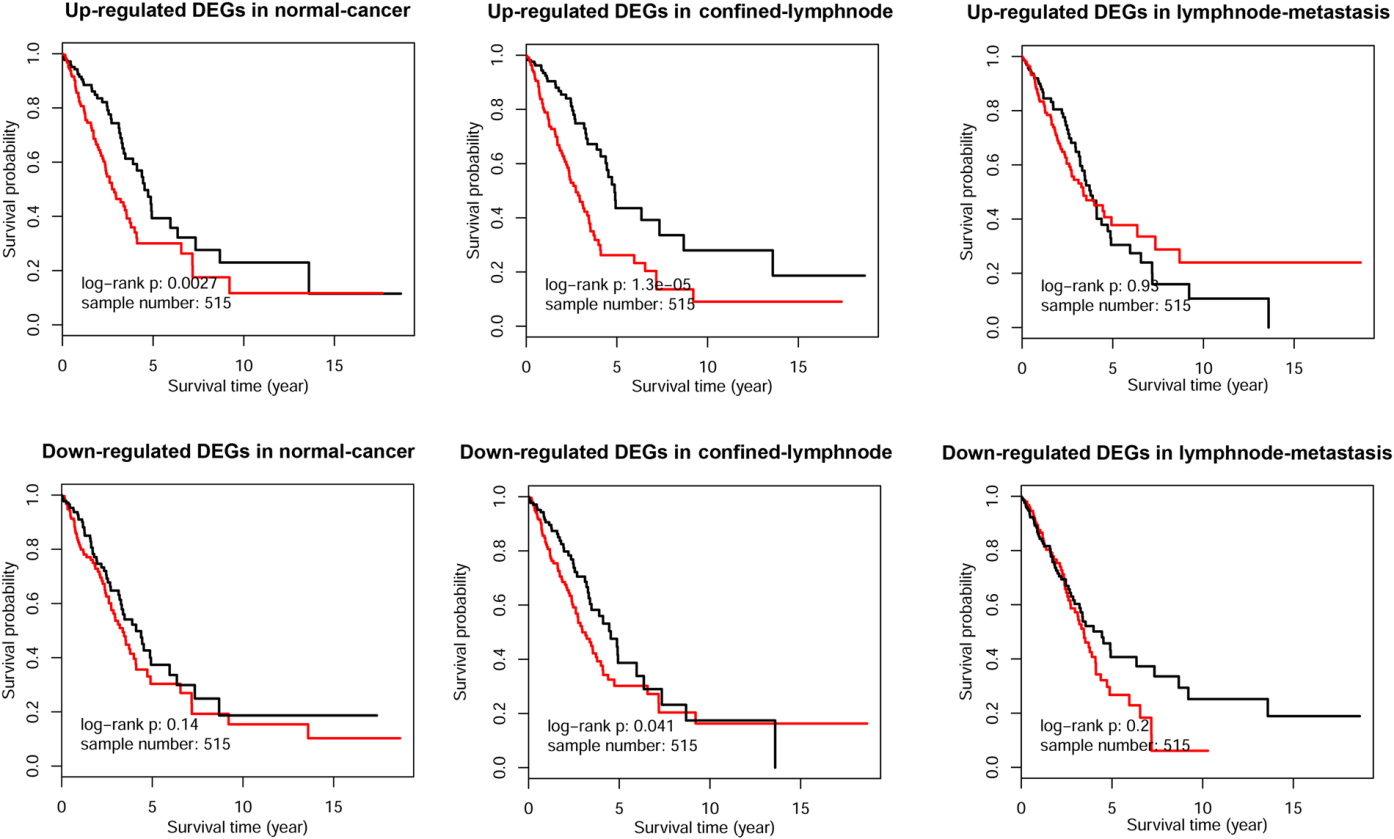
Survival analysis of corresponding significant DEGs in three transitions.

Kaplan–Meier survival analysis was conducted to evaluate the prognostic values of collected up-regulated and down-regulated DEGs during the three transitions, respectively. As shown in Fig. 5, lymphnode-metastasis DEGs were not significantly associated with OS. Up-regulated DEGs in normal-cancer transition, rather than down-regulated ones, were significant correlated with patient’s OS (*n* = 515, *p* = 0.0027). However, both up-regulated and down-regulated DEGs in confined-lymphnode transition were significant (*p* = 1.3e-5 and *p* = 0.041, respectively), suggesting the strongest association with LUAD’s prognosis.

### Biggest connected component composed of confined-lymphnode DEGs were intimately associated with patient’s OS and clinicopathological variables

**Figure 6:**
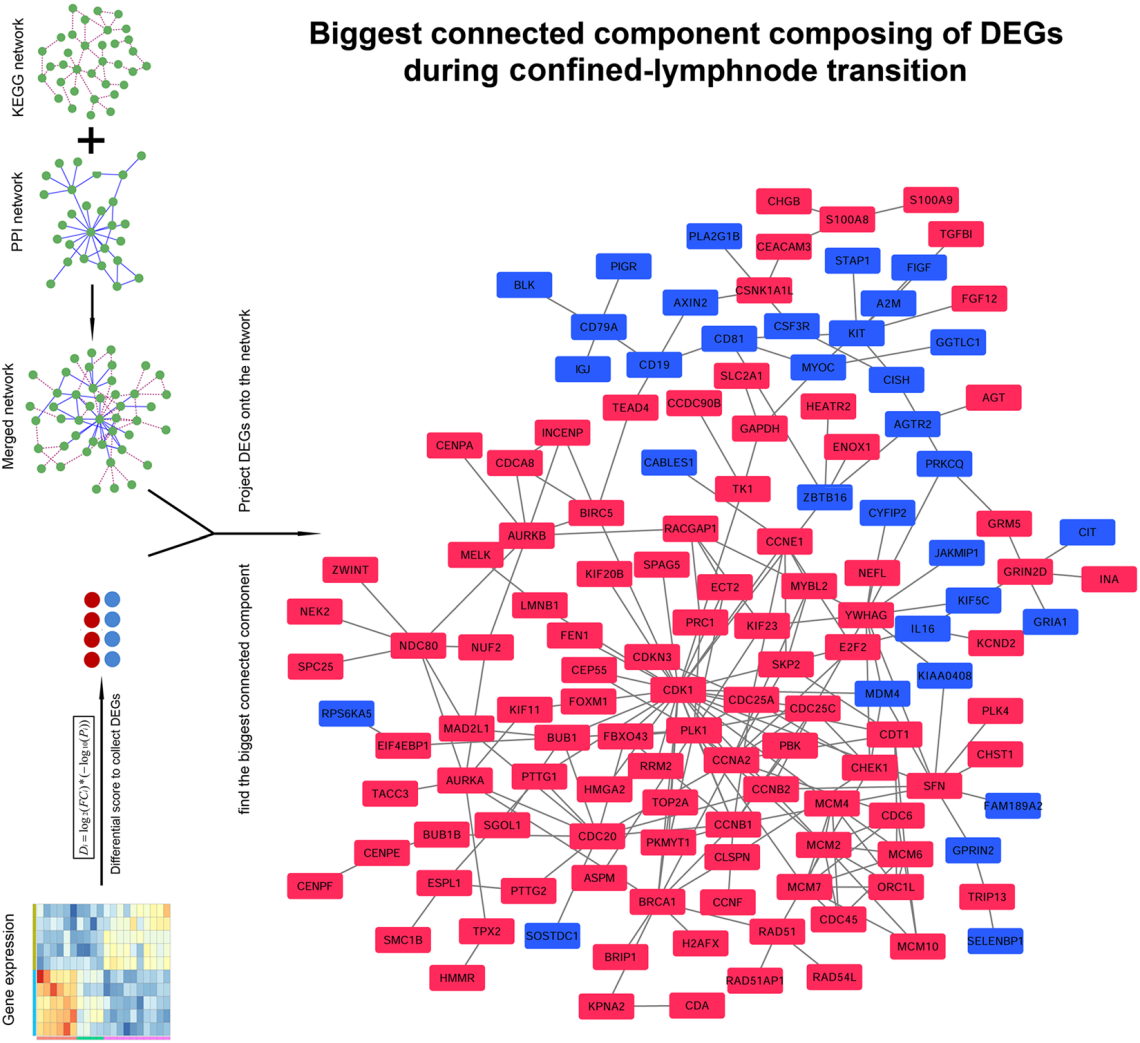
Retrieval of biggest connected module composed of DEGs during confined-lymphnode transition. Significant DEGs identified from confined-lymphnode transition were projected onto the merged biological network, and the biggest connected component was obtained for further analysis. Red nodes represented up-regulated DEGs, while blue ones represented down-regulated DEGs.

**Figure 7:**
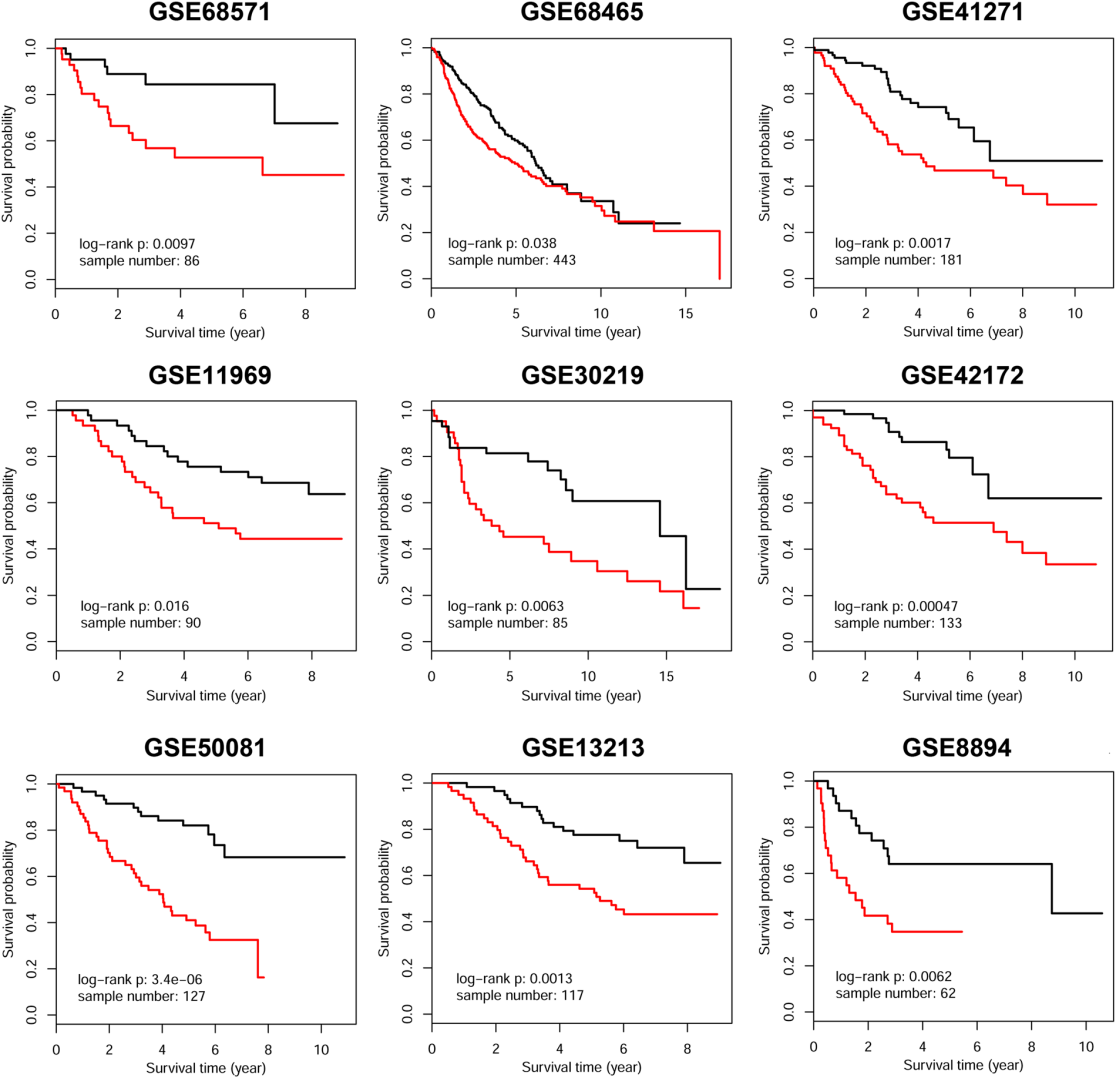
Survival analysis of 135 module DEGs in nine independent datasets.

We projected 1077 confined-lymphnode DEGs onto HPRD-KEGG merged biological network, and obtained the biggest connected component containing 135 DEGs (including 102 up-regulated and 33 down-regulated DEGs, Fig. 6). Nine LUAD microarray datasets were downloaded from GEO database in order to evaluate the prognostic value of these genes. The result indicated that DEGs within this connected module were significantly associated with patient’s OS in all 9 datasets (Fig. 7). To show the robustness of this methodology, we re-analyze these confined-lymphnode DEGs in a curated human singling network (HSN, http://www.cancer-systemsbiology.org/data-software, Supplementary Figures S2 and 3), and HPRD-KEGG-HSN merged network (Supplementary Figures S4 and 5), and the results were all satisfactory, indicating that the whole methodology in finding prognostic genes were quite stable and convincing.

Furthermore, we used 7 GEO datasets with definite information of OS, age, sex, stage or grade to evaluate the independence of the prognostic factors with Cox regression analysis in a stepwise manner (Table 1). The result indicated that these 135 connected genes were all significantly associated with patient’s OS in both univariate and multivariate Cox analysis (Table 1).

**Table 1:** Univariate and multivariate analyses of LUAD patient’s survival (Cox proportional hazards regression model) in 7 testing cohorts.

Factors	Univariate Cox regression		Multivariate Cox regression	
	*HR* (95% *CI*)	*p*	*HR* (95% *CI*)	*p*
***GSE13213***				
Age	1.006 (0.978~1.035)	0.678	—	—
Sex (Male/Female)	1.359 (0.773~2.387)	0.286	—	—
Stage (II + III/I)	2.536 (1.446~4.448)	**0.001**	2.1230 (1.200~3.781)	**0.010**
PC1^a^	0.374 (0.206~0.680)	**0.001**	0.435 (0.236~0.800)	**0.007**
***GSE50081***				
Age	1.020 (0.990~1.050)	0.192	—	—
Sex (Male/Female)	1.4101 (0.807~2.463)	0.228	—	—
Stage (II/I)	2.443 (1.383~4.316)	**0.002**	2.071 (1.167~3.676)	**0.013**
PC1^a^	0.263 (0.142~0.487)	**2.241e-05**	0.285 (0.153~0.532)	**7.867e-05**
***GSE42172***				
Age	1.043 (1.008~1.079)	**0.015**	1.037 (1.002~1.074)	**0.039**
Sex (Male/Female)	1.814 (0.968~3.398)	0.063	—	—
Stage (II + III + IV/ I)	2.029 (1.115~3.694)	**0.021**	1.346 (0.711~2.546)	0.361
PC1^a^	0.299 (0.150~0.596)	**5.998e-04**	0.321 (0.158~0.650)	**0.002**
***GSE30219***				
Age	1.038 (1.003~1.073)	**0.030**	1.050 (1.013~1.087)	**0.007**
Sex (Male/Female)	1.023 (0.492~2.127)	0.951	—	—
Stage (II + III/I)	1.003 (0.414~2.427)	0.995	—	—
PC1^a^	2.436 (1.291~4.595)	**0.006**	2.829 (1.481~5.405)	**0.002**
***GSE11969***				
Age	1.004 (0.972~1.038)	0.788	—	—
Sex (Male/Female)	1.332 (0.714~2.486)	0.367	—	—
Stage (II + III/I)	2.691 (1.427~5.075)	**0.002**	2.677 (1.418~5.052)	**0.002**
PC1^a^	2.156 (1.134~4.098)	**0.019**	2.140 (1.125~4.071)	**0.020**
***GSE41271***				
Age	1.018 (0.993~1.044)	0.154	—	—
Sex (Male/Female)	1.629 (1.004~2.643)	**0.048**	1.324 (0.802~2.185)	0.273
Stage (II + III + IV/I)	2.3581 (1.453~3.828)	**5.205e-4**	2.014 (1.222~3.318)	**0.006**
PC1^a^	0.459 (0.278~0.756)	**0.002**	0.579 (0.342~0.981)	**0.042**
***GSE68465***				
Age	1.026 (1.013~1.040)	**1.172e-04**	1.028 (1.015~1.042)	**4.514e-05**
Sex (Male/Female)	1.425 (1.098~1.848)	**0.008**	1.336 (1.027~1.738)	**0.031**
Grade (II + III/I)	1.181 (0.790~1.766)	0.418	—	—
PC1^a^	1.401 (1.081~1.817)	**0.011**	1.433 (1.100~1.867)	**0.008**

^a^Based on the median of the PC1 value to divide samples into two groups. Significant *p* values were in bold (*p* < 0.05). Abbreviations: *HR*, hazard ratio; *CI*, confidence interval.

We also compared this 135-gene signature to three other published LUAD prognostic signatures through Kaplan–Meier survival analysis and Cox analysis in these microarray independent datasets^21–23^. The result indicated that none of the other three signatures was statistically significant for all these independent datasets in both Kaplan–Meier analysis and Cox analysis, highly suggesting the validity and stability of this 135-gene signature (Supplementary Figures S6–8, Supplementary Tables S1–3).

## Discussion

Since carcinogenesis is a highly complicated biological process, studies of underlying molecular mechanisms in various types of cancers have always been the highlighted spot in cancer research. The precious knowledge we obtained from previous cancer studies undoubtedly provided us with the effective therapeutic methods to treat cancer patients. For instance, emerging molecular targeted drugs ushered us into a brand new era of clinical oncology^24–27^. However, the large scale heterogeneity of cancer cells succeeds in keeping us from fully understanding its intricate molecular mechanism. Cancer cells need to acquire functional capabilities to survive, proliferate, disseminate and colonize in distant organs. These functions are acquired in different tumor types via activating distinct hallmark networks and at various times during the course of multistep tumorigenesis^28^. Therefore, it is desirable to analyze the cancer hallmark genes from the DEGs and discover stable gene signatures with profound prognostic information^29^. In numerous cancer studies, the identification of differential expressed molecules is the first step in the identification of prognostic indicators. For instance, *AHNAK2* expression was identified as upregulated in clear cell renal cell carcinoma based on the comparison between cancer and adjacent normal tissues, leading to further exploration of its prognostic relevance^30^. The under-expression of lncRNA *LINC00261* was also first identified in gastric cancer comparing with normal adjacent tissues, was then proven associated with poor prognosis by promoting distant metastasis^31^. Admittedly, molecules undergoing substantial differentiation probably play important roles during carcinogenesis, and furthermore influence cancer patient’s survival accordingly. However, the samples for identifying differentially expressed molecules were, in most circumstances, majorly cancer and normal samples. This comparison is actually so plausible, that it has become the safest and most acceptable way in molecular cancer research^32–36^. However, expression differentiation between normal and cancer tissues is intended to measure the molecular dysregulations between the two extreme end points of carcinogenesis, and this oversimplified version of comparison probably overlooks the subtle molecular dysregulations happening during specific carcinogenic stage, for instance, metastasis. Therefore, identification of DEGs during the process of metastasis might uncover the intricate underlying molecular mechanism, and be greatly helpful in finding prognostic factors and potential therapeutic targets.

TCGA database is an immeasurable source of knowledge launched in 2005, which provides publicly available cancer genomic datasets^37^. With the aid of TCGA database, we successfully retrieved the RNA sequencing data of LUAD samples. Since the metastasis is so important in the carcinogenesis, we started to focus on the expression differentiation during this crucial biological process. Primary tumors were divided into three groups, i.e. *confined*, *lymphnode* and *metastasis*, to simulate the trajectory of metastasis, since lymph node invasion under most circumstances is the first step of tumor metastasis in LUAD. The reason why we used primary tumors in this study is to avoid systematic bias. Apparently, if the secondary metastatic tumors were used for DEG identification, tremendous systematic bias might be introduced since the molecular characteristics of the secondary site (including lymph nodes and distant organs) are surely different than primary sites, even in physiological state. Since the comparison between cancer samples and corresponding adjacent normal tissues is frequently used in molecular cancer research, LUAD paired samples were also extracted as the standard to evaluate the prognostic information of DEGs identified during metastatic process.

Not surprisingly, traditional *t* test could not identify significant DEGs during confined-lymphnode and lymphnode-metastasis transitions, due to relatively much less expression differentiation comparing with normal-cancer transition (Fig. 1). This difference in DEG numbers is quite easy to understand. The pathological characterization is completely distinct between normal and cancer, certainly as well as the molecular characteristics. Additionally, expression differentiations during confined-lymphnode and lymphnode-metastasis were inclined to concentrate upon a very small time window during the process of metastasis, and the pathological characteristics were almost the same for these primary tumors. In order to identify potential DEGs with subtle expression differentiation, we proposed a more “lenient” manner, differential score, as a new statistic alternative to quantify the molecular dysregulation. Based on differential score, top DEGs in both directions were collected, and the Cox correlation coefficients were calculated to represent corresponding prognostic values. The results consistently showed that DEGs during confined-metastasis transition were most closely related to patient’s OS (Fig. 2). As addressed in our previous study, a variety of molecules have already been differentially expressed in precancerous stage^38^. In this case, confined-lymphnode transition could be regarded as pre-metastatic stage, probably containing most precious untapped resource of prognostic information.

Based on extensive literature searching, the overexpression of the molecules up-regulated in cancer, in most of cancer studies, might shorten patient’s survival, while that of down-regulated ones might have the opposite effect. This impression is certainly not explicitly explained by any researchers, but the mainstream voice strongly advocates this research pattern, since nearly no exception has been ever found. In the scenario of LUAD, this is probably true. The means of Cox correlation coefficients in normal-cancer and confined-lymphnode transition were both accordant with the direction of expression differentiation, that is, up-regulated DEGs were associated with a poor prognosis, while down-regulated ones predicted a better prognosis. Additionally, the density curve of confined-lymphnode DEGs seemed gradually separated from the other two curves as the gene number was decreasing, suggesting DEGs identified form confined-lymphnode transition were probably most closely related to patient’s OS. Therefore, as mentioned before, comparison between *confined* and *lymphnode* primary tumors might be superior in survival association than traditional normal-cancer comparison.

Furthermore, significant DEGs were identified in each transition based on differential score in corresponding normal distribution, which was confirmed by Kolmogorov-Smirnov test. Although the expression differentiation in metastatic stages were not as distinct as in normal-cancer transition (Fig. 4A), the down-regulated and up-regulated DEGs could generally clustered into two groups, indicating the validity of our identification methodology. GSEA analysis indicated that DEGs in confined-lymphnode transition was significantly enriched in normal-cancer paired data, while lymphnode-metastasis DEGs were reversely enriched with a lower enrichment score, suggesting expression differentiation status was generally consistent between normal-cancer and confined-lymphnode transitions (Fig. 4B). Moreover, the survival analysis further confirmed that both up-regulated and down-regulated DEGs during confined-lymphnode transition were significantly related to patient’s OS (Fig. 5). Based on our aforementioned findings, DEGs during confined-lymphnode transition showed similar molecular characteristics with normal-cancer transition, whereas contained much more prognostic information, indicating confined-lymphnode transition model might be more helpful in potential clinical applications for cancer patients.

In order to identify the functional module undergoing tremendous expression differentiation during confined-lymphnode transition, the biggest connected component composed of DEGs in this transition was retrieved (*n* = 135, Fig. 6). In this connected module, these two groups of genes separately formed compact regulatory interactions, and many regulations existed between the two gene groups. As mentioned in previous study, dynamic modularity analysis based on this network might help identify core events during carcinogenesis and genes that predict prognosis^39^. Because interpatient and intratumour heterogeneity has an important role in affecting the robustness of gene signatures^40^, therefore we check the robustness of the prognosis signatures based on 9 independent GEO datasets. The result indicated that the expression of these 135 module DEGs were all significantly associated with patient’s OS (Fig. 7). Therefore, this module, containing 102 up-regulated genes and 33 down-regulated genes subject to strong expression alterations during the early stage of metastasis, may represent an important mechanism of tumor invasion and migration.

In summary, expression profiles of adjacent normal tissues and three types of primary tumor samples (*confined*, *lymphnode*, and *metastasis*) of LUAD were downloaded to identify genes with prognostic value. Differential score was used in DEG identification, and the result indicated that significant DEGs during confined-lymphnode transition were most informative on the basis of patient’s prognosis. Taking advantage of public curated biological network, the largest connected module were obtained, and the expression of its 135 genes was significantly associated with patients’ OS. Hopefully these DEGs might be helpful in the discovery of prognostic factors and therapeutic targets for LUAD patients. In future researches, we attempt to quantify and computationally dissect clones from tumors and conduct clone-based analysis, in order to decipher the evolutionary dynamics of tumor clonal networks during confined-lymphnode transition^41, 42^. Additionally, circulating tumor cells (CTCs) and cell-free DNAs (cfDNAs) in blood samples could also be used to discover the promising biomarkers and promising therapeutic targets^43^. The underlying molecular mechanism during confined-lymphnode transition might surprise us with its great value in cancer research.

## Electronic supplementary material

Supplementary Figure S1-8 Table S1-3
