# Supplementary material for: Identification of prognostic genes through expression differentiation during metastatic process in lung adenocarcinoma
Source: Sci Rep. 2017 Sep 11;7:11119. doi: 10.1038/s41598-017-11520-6 (PMC5593941; doi:10.1038/s41598-017-11520-6)
Supplement: Supplementary file 1 — Supplementary Figure S1-8 Table S1-3 [file 41598_2017_11520_MOESM1_ESM.pdf]

Identification of prognostic genes through expression differentiation during metastatic process in lung adenocarcinoma

Ning An[1], Xue Yang[1,†]

[1] Department of Oncology, the Affiliated Hospital of Qingdao University, Qingdao， 266003, China.

[†] Correspondence and requests for materials should be addressed to X.Y. (yxue0409@outlook.com)

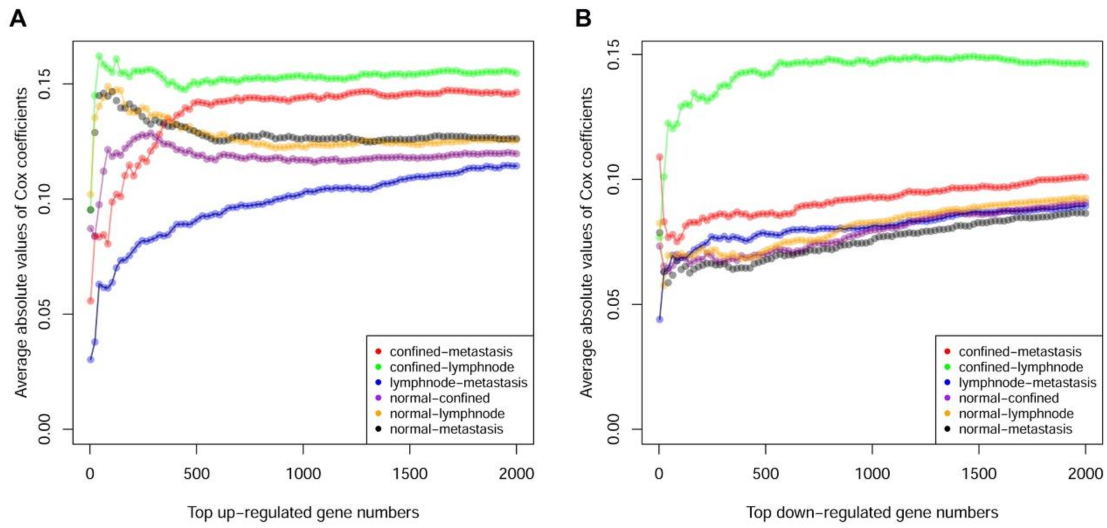

Supplementary Figure S1. AACCV-TGN analysis in six comparisons in both up-regulated (A) and down-regulated directions (B).

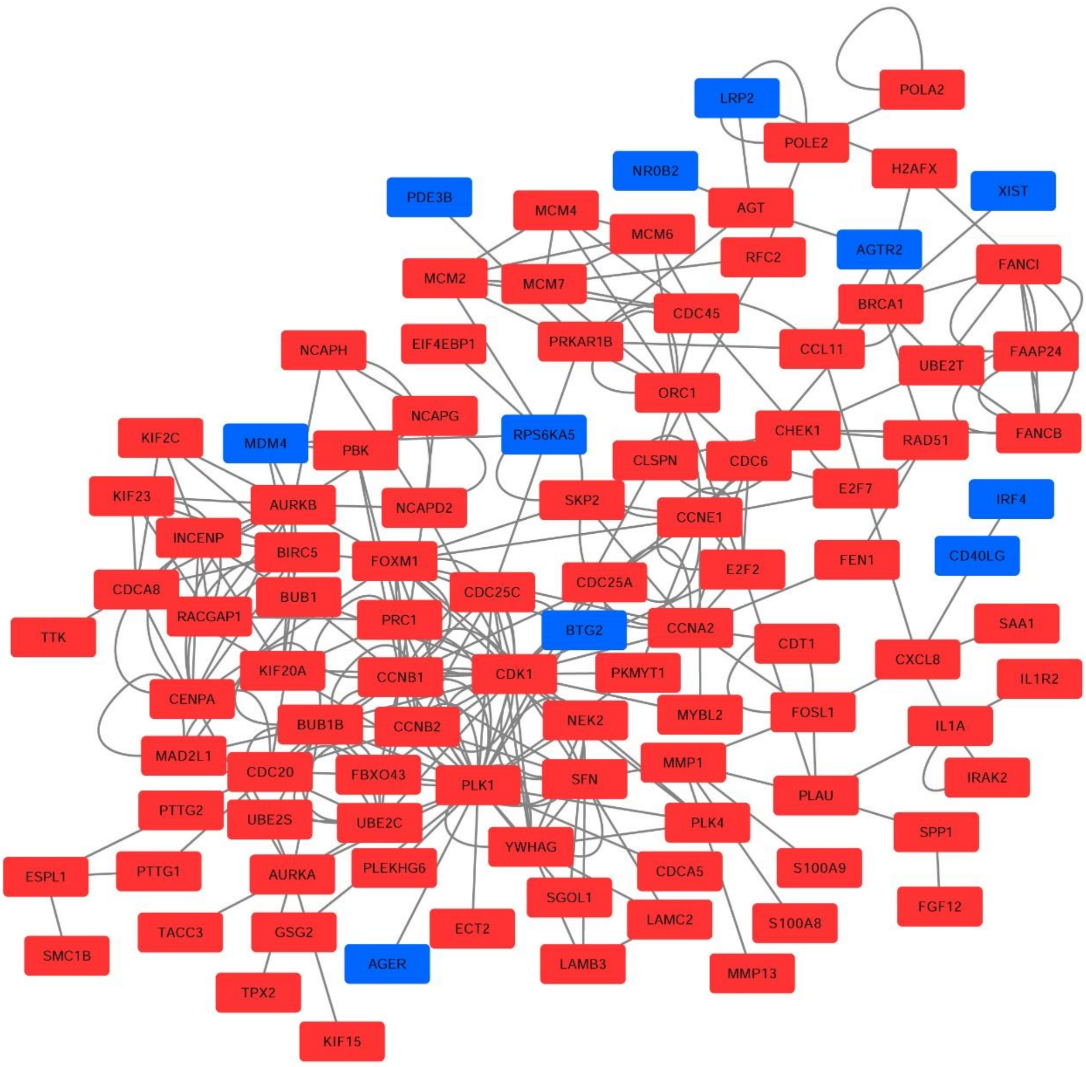

Supplementary Figure S2. Retrieval of biggest connected module composed of DEGs during confined-lymphnode transition in human singling network (HSN).

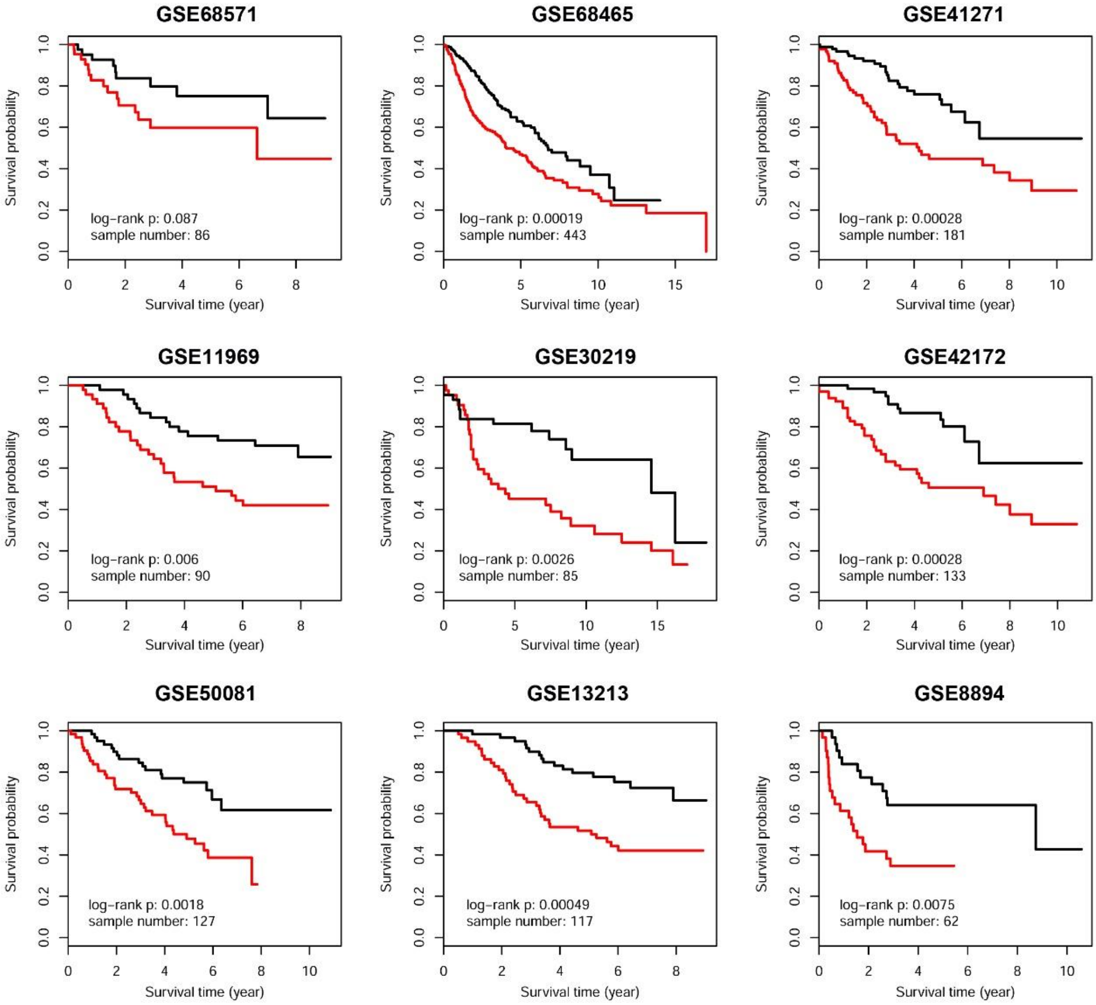

Supplementary Figure S3. Survival analysis of HSN module DEGs in nine independent datasets.

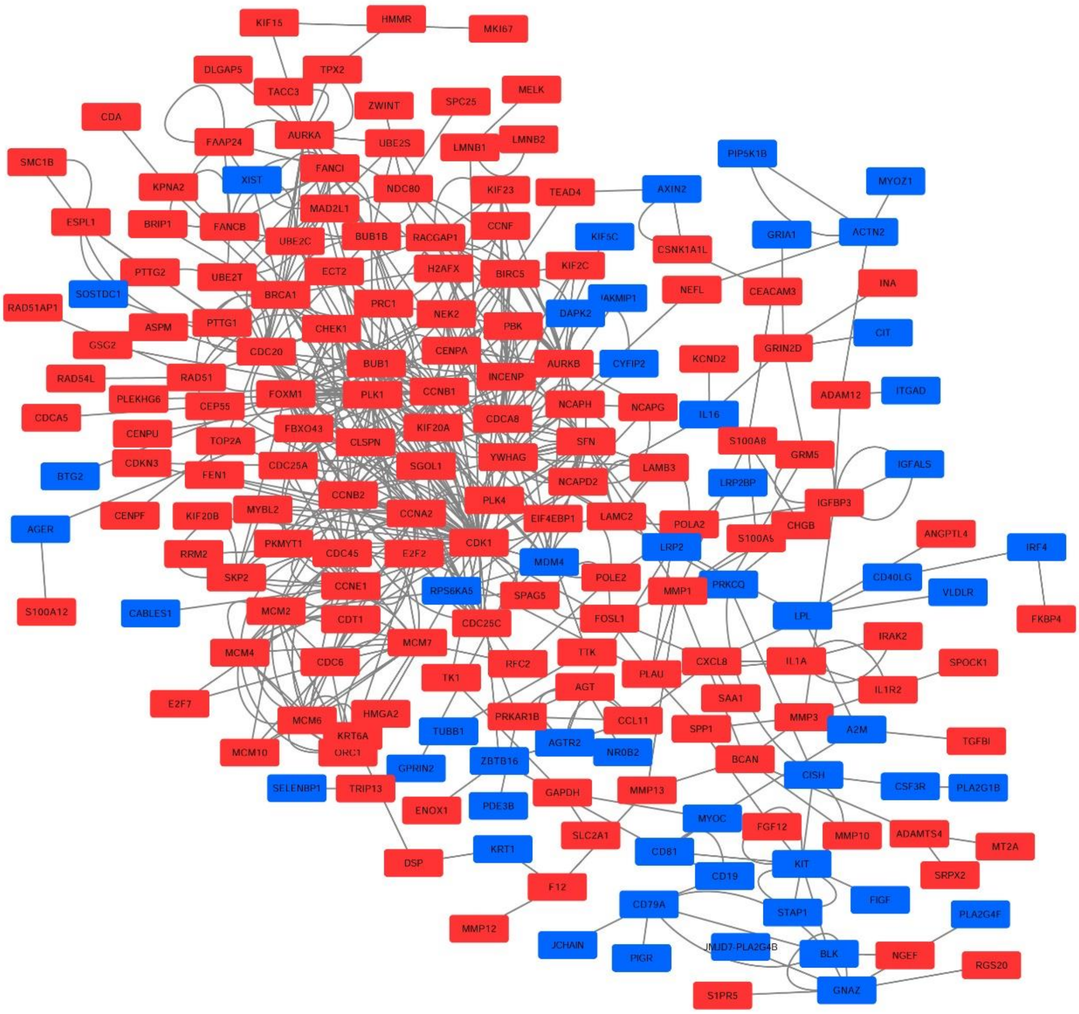

Supplementary Figure S4. Retrieval of biggest connected module composed of DEGs during confined-lymphnode transition in HPRD-KEGG-HSN merged network.

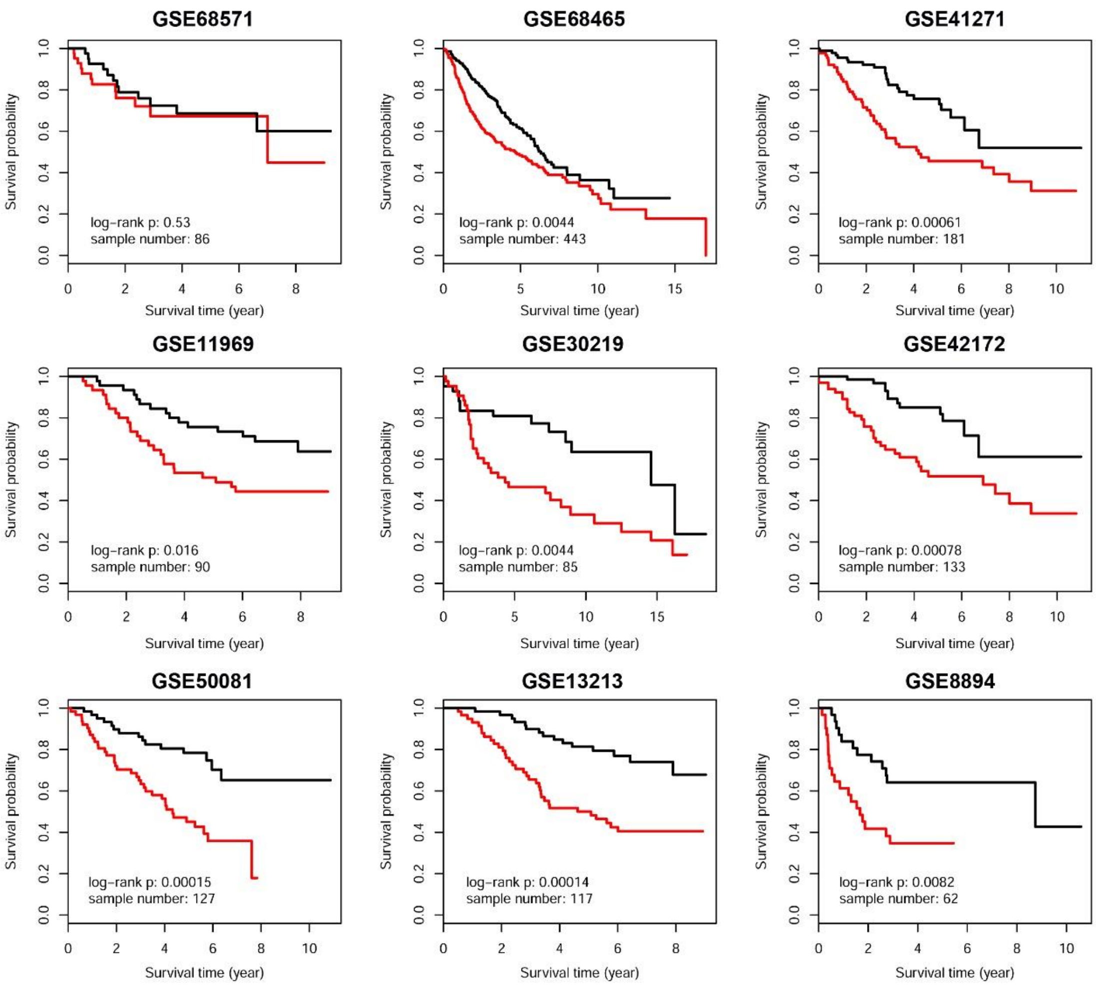

Supplementary Figure S5. Survival analysis of HPRD-KEGG-HSN module DEGs in nine independent datasets.

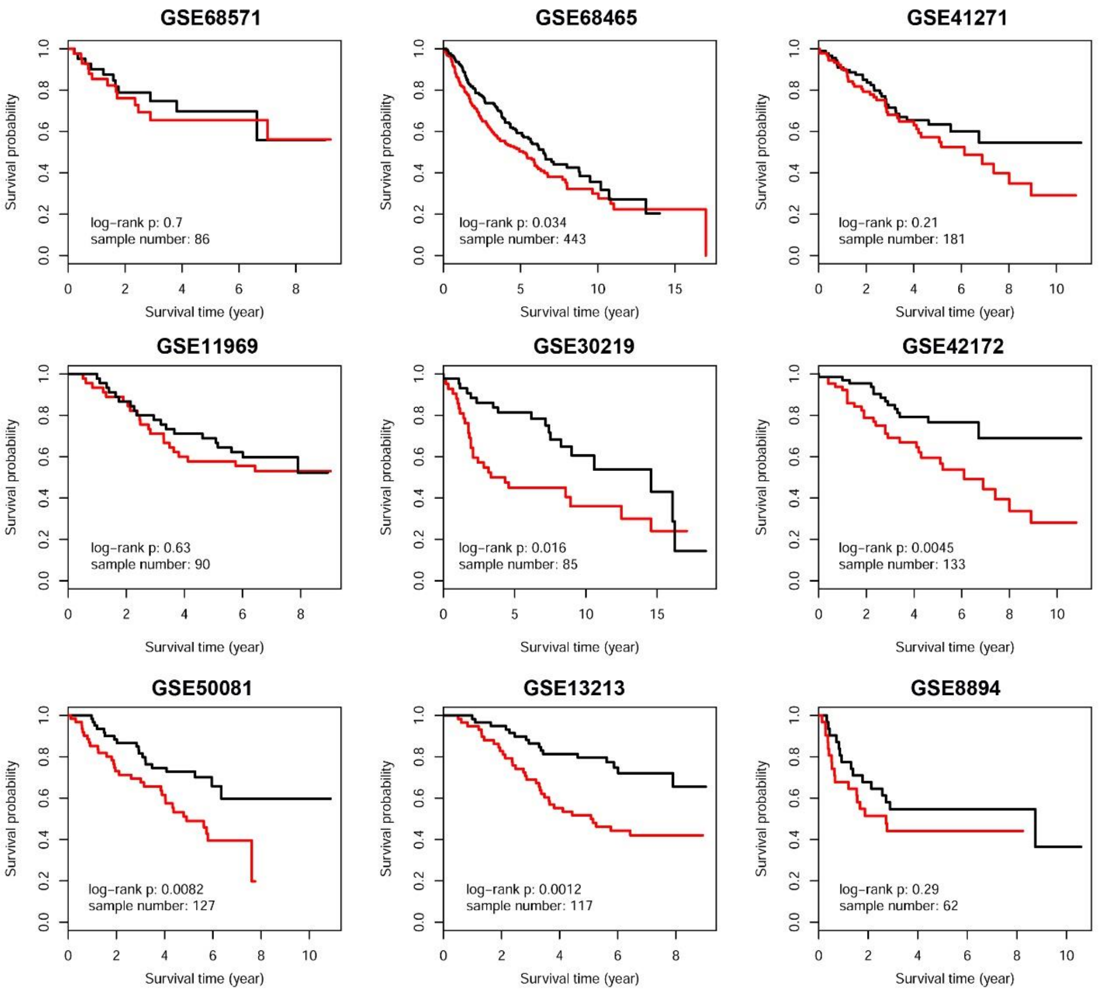

Supplementary Figure S6. Survival analysis of the gene signature proposed by Chen et.al. in nine independent datasets.

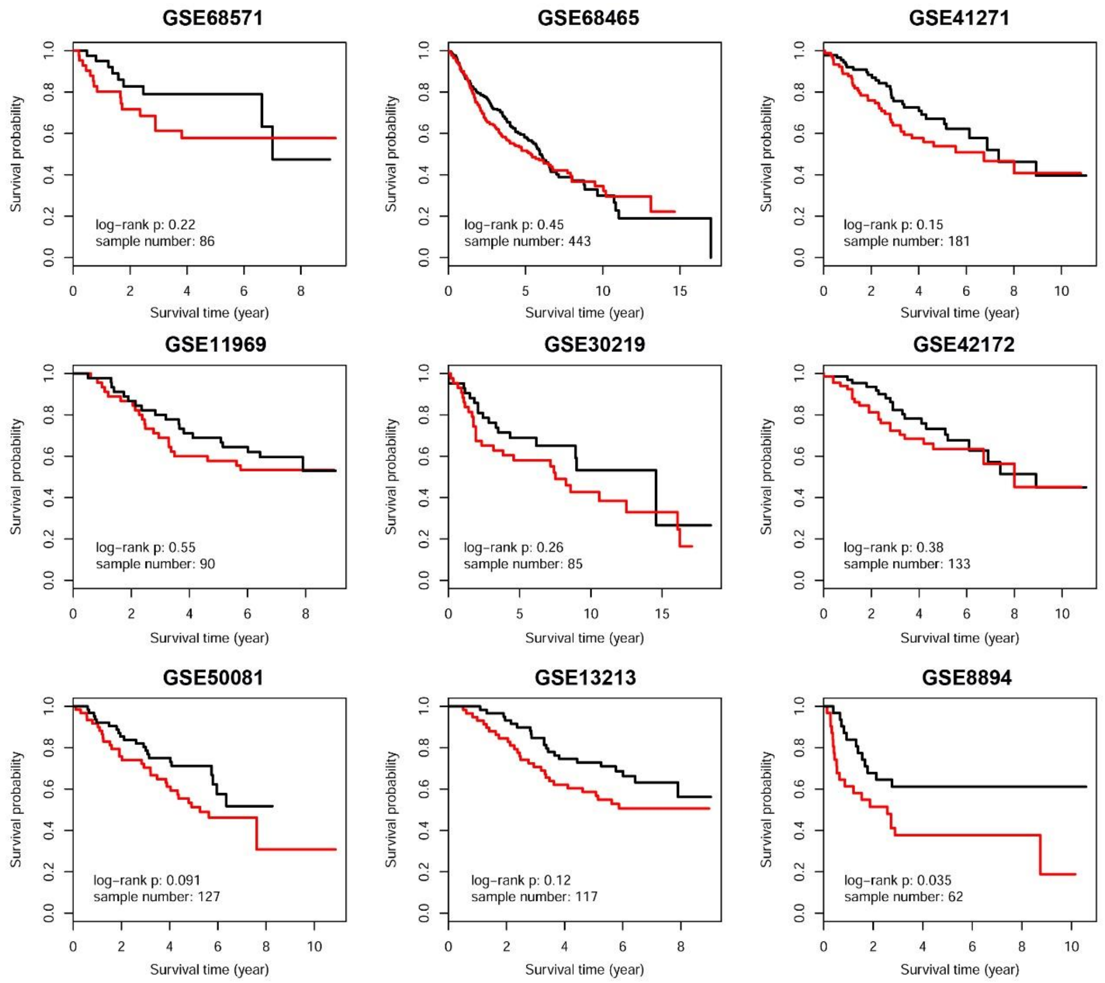

Supplementary Figure S7. Survival analysis of the gene signature proposed by Boutros et.al. in nine independent datasets.

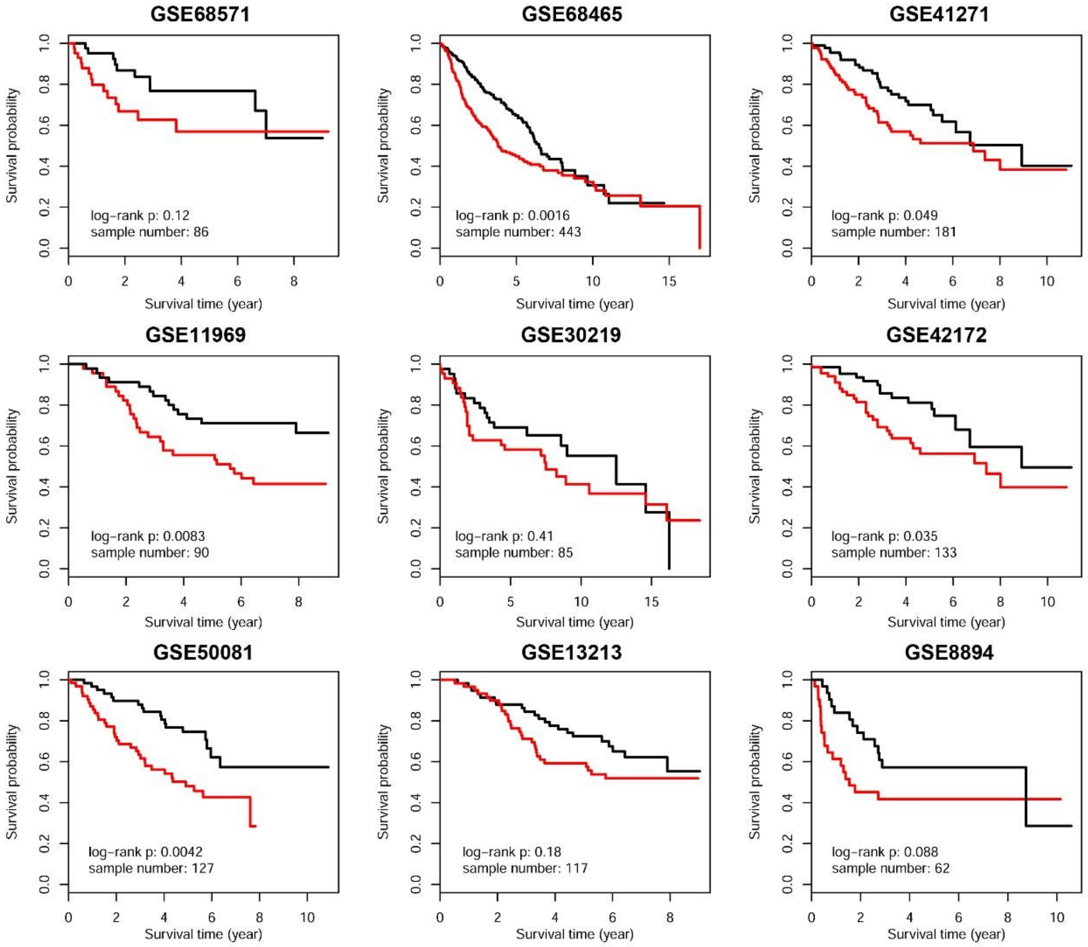

Supplementary Figure S8. Survival analysis of the gene signature proposed by Bianchi et.al. in nine independent datasets.

Factors	Univariate Cox regression		Multivariate Cox regression	
	HR **(95% *CI*)**	***p***	***HR*** **(95% *CI*)**	***p***
GSE13213				
Age	1.006 (0.978~1.035)	0.678	-	-
Sex (Male/Female)	1.359 (0.773~2.387)	0.286	-	-
Stage (II+III/I)	2.536 (1.446~4.448)	**0.001**	2.363 (1.345~4.150)	**0.003**
PC1[a]	0.387 (0.213~0.703)	**0.002**	0.413 (0.226~0.752)	**0.004**
GSE50081				
Age	1.020 (0.990~1.050)	0.192	-	-
Sex (Male/Female)	1.4101 (0.807~2.463)	0.228	-	-
Stage (II/I)	2.443 (1.383~4.316)	**0.002**	2.331 (1.317~4.125)	**0.004**
PC1[a]	0.512 (0.292~0.899)	**0.020**	0.540 (0.307~0.949)	**0.032**
GSE42172				
Age	1.043 (1.008~1.079)	**0.015**	1.030 (0.995~1.066)	0.091
Sex (Male/Female)	1.814 (0.968~3.398)	0.063	-	-
Stage (II+III+IV/ I)	2.029 (1.115~3.694)	**0.021**	1.498 (0.796~2.819)	0.210
PC1[a]	0.408 (0.215~0.772)	**0.006**	0.480 (0.248~0.928)	**0.029**
GSE30219				
Age	1.038 (1.003~1.073)	**0.030**	1.048 (1.011~1.085)	**0.010**
Sex (Male/Female)	1.023 (0.492~2.127)	0.951	-	-
Stage (II+III/I)	1.003 (0.414~2.427)	0.995	-	-
PC1[a]	1.972 (1.082~3.596)	**0.027**	2.285 (1.238~4.221)	**0.008**
GSE11969				
Age	1.004 (0.972~1.038)	0.788	-	-
Sex (Male/Female)	1.332 (0.714~2.486)	0.367	-	-
Stage (II+III/I)	2.691 (1.427~5.075)	**0.002**	-	-
PC1[a]	0.860 (0.462~1.601)	0.635	-	-
GSE41271				
Age	1.018 (0.993~1.044)	0.154	-	-
Sex (Male/Female)	1.629 (1.004~2.643)	**0.048**	1.502 (0.924~2.444)	0.101
Stage (II+III+IV/I)	2.3581 (1.453~3.828)	**5.205e-4**	2.264 (1.392~3.684)	**0.001**
PC1[a]	1.355 (0.842~2.182)	0.211	-	-
GSE68465				
Age	1.026 (1.013~1.040)	**1.172e-04**	1.027 (1.013~1.041)	**9.770e-05**
Sex (Male/Female)	1.425 (1.098~1.848)	**0.008**	1.392 (1.072~1.806)	**0.013**
Grade (II+III/I)	1.181 (0.790~1.766)	0.418	-	-
PC1[a]	0.749 (0.578~0.970)	**0.028**	0.735 (0.567~0.952)	**0.020**

Supplementary Table S1. Univariate and multivariate analyses of LUAD patient's survival (Cox proportional hazards regression model) in 7 testing cohorts with the gene signature proposed by Chen et.al.

Factors	Univariate Cox regression		Multivariate Cox regression	
	HR **(95% *CI*)**	***p***	***HR*** **(95% *CI*)**	***p***
GSE13213				
Age	1.006 (0.978~1.035)	0.678	-	-
Sex (Male/Female)	1.359 (0.773~2.387)	0.286	-	-
Stage (II+III/I)	2.536 (1.446~4.448)	**0.001**	-	-
PC1[a]	0.638 (0.362~1.124)	0.120	-	-
GSE50081				
Age	1.020 (0.990~1.050)	0.192	-	-
Sex (Male/Female)	1.4101 (0.807~2.463)	0.228	-	-
Stage (II/I)	2.443 (1.383~4.316)	**0.002**	-	-
PC1[a]	0.620 (0.355~1.084)	0.093	-	-
GSE42172				
Age	1.043 (1.008~1.079)	**0.015**	1.036 (1.000~1.073)	**0.049**
Sex (Male/Female)	1.814 (0.968~3.398)	0.063	-	-
Stage (II+III+IV/ I)	2.029 (1.115~3.694)	**0.021**	1.718 (0.928~3.182)	0.085
PC1[a]	0.736 (0.404~1.342)	0.317	-	-
GSE30219				
Age	1.038 (1.003~1.073)	**0.030**	-	-
Sex (Male/Female)	1.023 (0.492~2.127)	0.951	-	-
Stage (II+III/I)	1.003 (0.414~2.427)	0.995	-	-
PC1[a]	1.406 (0.769~2.567)	0.268	-	-
GSE11969				
Age	1.004 (0.972~1.038)	0.788	-	-
Sex (Male/Female)	1.332 (0.714~2.486)	0.367	-	-
Stage (II+III/I)	2.691 (1.427~5.075)	**0.002**	-	-
PC1[a]	0.829 (0.445~1.543)	0.554	-	-
GSE41271				
Age	1.018 (0.993~1.044)	0.154	-	-
Sex (Male/Female)	1.629 (1.004~2.643)	**0.048**	1.502 (0.924~2.444)	0.101
Stage (II+III+IV/I)	2.358 (1.453~3.828)	**5.205e-4**	2.264 (1.392~3.684)	**0.001**
PC1[a]	1.420 (0.882~2.286)	0.149	-	-
GSE68465				
Age	1.026 (1.013~1.040)	**1.172e-04**	1.026 (1.013~1.039)	**1.509e-4**
Sex (Male/Female)	1.425 (1.098~1.848)	**0.008**	1.408 (1.085~1.826)	**0.010**
Grade (II+III/I)	1.181 (0.790~1.766)	0.418	-	-
PC1[a]	1.097 (0.848~1.419)	0.483	-	-

Supplementary Table S2. Univariate and multivariate analyses of LUAD patient's survival (Cox proportional hazards regression model) in 7 testing cohorts with the gene signature proposed by Boutros et.al.

Factors	Univariate Cox regression		Multivariate Cox regression	
	***HR* (95% *CI*)**	***p***	***HR* (95% *CI*)**	***p***
GSE13213				
Age	1.006 (0.978~1.035)	0.678	-	-
Sex (Male/Female)	1.359 (0.773~2.387)	0.286	-	-
Stage (II+III/I)	2.536 (1.446~4.448)	**0.001**	-	-
PC1[a]	1.475 (0.837~2.600)	0.179	-	-
GSE50081				
Age	1.020 (0.990~1.050)	0.192	-	-
Sex (Male/Female)	1.4101 (0.807~2.463)	0.228	-	-
Stage (II/I)	2.443 (1.383~4.316)	**0.002**	2.246 (1.268~3.979)	**0.006**
PC1[a]	0.410 (0.230~0.729)	**0.002**	0.439 (0.246~0.783)	**0.005**
GSE42172				
Age	1.043 (1.008~1.079)	**0.015**	1.032 (0.996~1.069)	0.078
Sex (Male/Female)	1.814 (0.968~3.398)	0.063	-	-
Stage (II+III+IV/ I)	2.029 (1.115~3.694)	**0.021**	1.709 (0.920~3.175)	0.090
PC1[a]	0.486 (0.259~0.912)	**0.025**	0.520 (0.277~0.978)-	**0.043**
GSE30219				
Age	1.038 (1.003~1.073)	**0.030**	-	-
Sex (Male/Female)	1.023 (0.492~2.127)	0.951	-	-
Stage (II+III/I)	1.003 (0.414~2.427)	0.995	-	-
PC1[a]	1.286 (0.707~2.340)	0.410	-	-
GSE11969				
Age	1.004 (0.972~1.038)	0.788	-	-
Sex (Male/Female)	1.332 (0.714~2.486)	0.367	-	-
Stage (II+III/I)	2.691 (1.427~5.075)	**0.002**	2.437 (1.284~4.622)	**0.006**
PC1[a]	2.346 (1.222~4.505)	**0.010**	2.074 (1.075~4.002)	**0.030**
GSE41271				
Age	1.018 (0.993~1.044)	0.154	-	-
Sex (Male/Female)	1.629 (1.004~2.643)	**0.048**	1.370 (0.828~2.267)	0.220
Stage (II+III+IV/I)	2.358 (1.453~3.828)	**5.205e-4**	2.211 (1.358~3.600)	**0.001**
PC1[a]	0.598 (0.369~0.969)	**0.037**	0.704 (0.426~1.162)	0.170
GSE68465				
Age	1.026 (1.013~1.040)	**1.172e-04**	1.027 (1.013~1.041)	**9.208e-5**
Sex (Male/Female)	1.425 (1.098~1.848)	**0.008**	1.321 (1.015~1.719)	**0.038**
Grade (II+III/I)	1.181 (0.790~1.766)	0.418	-	-
PC1[a]	1.556 (1.199~2.019)	**8.810e-4**	1.519 (1.167~1.978)	**0.002**

Supplementary Table S3. Univariate and multivariate analyses of LUAD patient's survival (Cox proportional hazards regression model) in 7 testing cohorts with the gene signature proposed by Bianchi et.al.
